# Nanoparticles Functionalised with Re(I) Tricarbonyl Complexes for Cancer Theranostics

**DOI:** 10.3390/ijms22126546

**Published:** 2021-06-18

**Authors:** Marcus Mkhatshwa, Joshua Mamolatelo Moremi, Katlego Makgopa, Amanda-Lee Ezra Manicum

**Affiliations:** Department of Chemistry, Faculty of Science, Tshwane University of Technology (Arcadia Campus), Pretoria 0001, South Africa; mkhatshwathedreamer@gmail.com (M.M.); joshmolatelo2@gmail.com (J.M.M.)

**Keywords:** cancer, nanoparticles, iron oxide, rhenium(I) tricarbonyl, nanotheranostic.

## Abstract

Globally, cancer is the second (to cardiovascular diseases) leading cause of death. Regardless of various efforts (i.e., finance, research, and workforce) to advance novel cancer theranostics (diagnosis and therapy), there have been few successful attempts towards ongoing clinical treatment options as a result of the complications posed by cancerous tumors. In recent years, the application of magnetic nanomedicine as theranostic devices has garnered enormous attention in cancer treatment research. Magnetic nanoparticles (MNPs) are capable of tuning the magnetic field in their environment, which positively impacts theranostic applications in nanomedicine significantly. MNPs are utilized as contrasting agents for cancer diagnosis, molecular imaging, hyperfusion region visualization, and T cell-based radiotherapy because of their interesting features of small size, high reactive surface area, target ability to cells, and functionalization capability. Radiolabelling of NPs is a powerful diagnostic approach in nuclear medicine imaging and therapy. The use of luminescent radioactive rhenium(I), ^188/186^Re, tricarbonyl complexes functionalised with magnetite Fe_3_O_4_ NPs in nanomedicine has improved the diagnosis and therapy of cancer tumors. This is because the combination of Re(I) with MNPs can improve low distribution and cell penetration into deeper tissues.

## 1. Introduction

**Figure 1:**
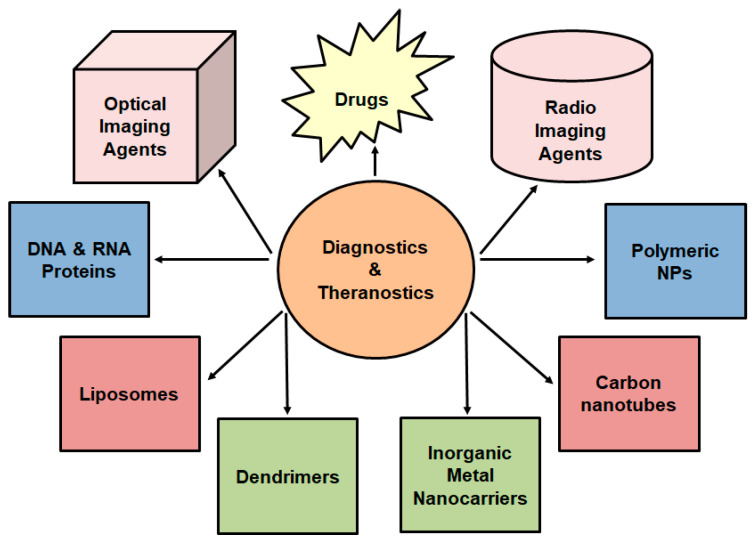
A schematic representation of nanotheranostics used for simultaneous release and imaging.

Cancer is a well-known, complicated and multistage disease caused by an uncontrolled division of abnormal cells in the body [1]. Regardless of the continuous progress in cancer diagnosis and therapy, this disease remains the second leading cause of death globally [2]. As much as the conventional cancer treatment approaches (i.e., surgery, radiotherapy, and chemotherapy) have shown positive impacts on cancer mortality rate, there still exist several challenges in cancer management. Amongst the stated treatment approaches, radiotherapy displays an added advantage as patients treated from this approach exhibit an improved long-term survival. Radiotherapy is a cancer treatment approach, falling under the umbrella of nuclear medicine, utilizing high doses of radiation to kill cancer cells and reduce the size of tumors [3]. Nuclear medicine, also known as radiopharmaceuticals, involves the use of radioisotopes bound to biological molecules that are capable of targeting specific organs, tissues, or cells. This field of medicine has been broadly studied as an advanced diagnostic tool where radionuclides are introduced in vivo. This is followed by the detection of the emitted gamma rays and generation of images which give detailed radionuclides distribution as well as physiological characterization of targeted areas [4,5]. An emerging area in nuclear medicine incorporates nano-imaging agents (see Figure 1) with dual behavior as both diagnostic and therapeutic tools [6,7]. However, due to the low distribution and cell penetration of these nanomaterials, their undesired pharmacokinetics had to be improved [8]. Thus, different nanotheranostics based on polymeric NPs have been manufactured and radiolabeled with available radionuclides of choice [9]. Within these polymeric NPs, various techniques are utilized to diagnose and treat cancerous diseases [10,11,12]. Theranostic nanomedicine, also known as nanotheranostics, involves treatments with nanosize particles (<100 nm) and has a large number of capabilities such as targeted delivery, controlled release, greater transport efficiency via endocytosis, stimuli-responsive systems, and the combination of therapeutic approaches such as multimodality diagnosis and therapy [13,14].

Subsequently, nanotheranostics’ stability has been improved by linking molecules such as chelator agents that can bind to radionuclides (i.e., ^186^Re, ^188^Re, and ^99m^Tc) and NPs [15]. The radioactive properties of these radionuclides are shown in Table 1.

Radionuclides which solely emit gamma rays (γ) such as ^99m^Tc possess diagnostic purposes, while radionuclides such as ^186^Re and ^188^Re emit beta particles (β) and gamma rays (γ) for therapeutic and diagnostics purposes, respectively. This ultimately led to the introduction of the Re(I) tricarbonyl core in the theranostic application by Alberto et al. (1999) [16] and Top et al. (1995) [17]. In their studies, they participated in the group VIIB transitional metal chemistry via the synthesis of a facile method to yield the [M(CO)_3_]^+^ core, where M = Tc or Re. Most importantly, the low-spin *d*^6^ electronic configuration and the stability of the CO ligands make the substitution in the [Tc or Re(CO)_3_]^+^ core useful in radiopharmaceutical chemistry [18]. From these two metal cores, the Re(I) tricarbonyl core displays an added advantage, since its chemistry can be studied with this metal being in a natural state as opposed to the radioisotopic state. Moreover, the biological application of the relatively small size Re(I) tricarbonyl moiety as compared to the kinetic stability and inertness, serves as a potential advantage [19,20]. Additionally, the kinetically inert Re(I) tricarbonyl complexes exhibit distinct phosphorescence/luminescence properties, depending on the nature of the ligands. This is another reason they found a huge application as photosensitizers and bio-imaging agents [21]. Conversely, the use of SPION layered material with radionuclides as theranostics provides great potential to improve the delivery processes of radionuclides into the targeted tissues. In this review, the main focus is based on the class of hybrid MRI-OI probes that are made by utilizing ultra SPION and sensitized luminescence compounds with the *d*-block element, Re. The potential applications of the nanoparticles (i.e., magnetite Fe_3_O_4_) functionalised with the Re(I) tricarbonyl complexes as a bimodal contrast agent for MRI and optical imaging of nanoparticles have been demonstrated by Carron et al., 2015 [22].

## 2. Magnetic Nanoparticles

Magnetic NPs have brought enormous attention to several biomedical applications, due to their intrinsic biocompatibility and interaction with externally applied magnetic fields. This is because magnetic NPs can distort magnetic fields in their surroundings, which establishes the basis for intensified contrast in MRI. In other uses, the applied magnetic fields can create magnetic forces and torques on their magnetic dipoles, leading to particle translation, rotation, and even energy dissipation in the form of heat. This phenomenon results in applications in magnetic biomarker or cell break-up targeted drug delivery, magneto-mechanical actuation of cell surface receptors, magnetic hyperthermia and triggered drug release as well as biomedical imaging. That is why there has been fast-growing research based on the synthesis, characterization, and post-synthesis application-specific to modification of magnetic Fe_3_O_4_ and substituted ferrite nanoparticles. This has led to several emerging uses in a broad array of fields such as medical and biomedical applications [23,24,25,26]. Thus, this review focuses on the coordinated Re(I) tricarbonyl complexes, functionalized with the magnetic Fe_3_O_4_ NPs as MRI-OI probes.

### 2.1. Iron Oxide NPs for Biomedical Applications

Several materials and compositions have been utilized to compose magnetic NPs by differentiating magnetic and physical properties necessary for the intended use. Still, in the biomedical arena, the potential biocompatibility and the long-term in vivo fate and clearance of magnetic NPs must be taken into account. These accountabilities prohibit the nanoparticle compositions and formulations that can be applied safely without presenting harm or side effects to living tissue [27,28]. This makes a specific subgroup of ferrite nanoparticles, M_x_Fe_3_-xO_4_ (M = Fe, Mn, Ni, or Zn; x = divalent cation) the best candidates for biomedical uses [29,30]. The biocompatibility of magnetic iron oxide NPs is less of a concern because a healthy human body already has mechanisms for handling, storage, and the use of iron [31]. Iron is an essential nutrient to sustain human health and survival. Essentially, it participates in the transport and storage of oxygen throughout the body, DNA synthesis, energy production, and metabolism, and detoxification; thus, it acts as both an antioxidant and pro-oxidant. Generally, the average human body has about 4 g of iron, and smaller contents of the other metals, in the form of two highly significant molecules, ferritin and haemoglobin [32].

Secondly, there has been extensive testing concerning the safety of these nanoparticles in laboratory, preclinical, and clinical settings; this is why ferrite magnetic NPs are preferred over others for biomedical applications. Many formulations of iron oxide have been accepted by regulatory agencies in both the United States and Europe for clinical-stage examination and use. For instance, the treatment of pancreatic and brain tumors [33,34], their applications in imaging and diagnostic settings via magnetic resonance imaging (MRI), and their employment for sentinel lymph node (SLN) mapping [35].

### 2.2. Magnetic Resonance Imaging (MRI)

MRI is used to investigate the properties of magnetic NPs such as Fe_3_O_4_ and Fe_2_O_3_. When magnetic NPs are introduced, they generate a local magnetic field, which results in the disturbance of the nuclear relaxation of magnetic nuclei in the environment [36]. These NPs can further stimulate the relaxation process and shorten the relaxation time of neighboring protons, intensifying the signal contrast between the surroundings and distal background in MR images. Unfortunately, MRI applies contrast agents for imaging which can be demanding, because it requires an extra effort to identify and prepare suitable imaging agents for targeted application. However, the use of magnetic Fe_3_O_4_ NPs is advantageous because they are bio-compatible for in vivo applications [37].

### 2.3. Clinical Applications of SPIONs

SPIONs are utilized as iron supplements in anaemic patients due to their non-toxic and bio-compatible nature [38]. They are also being examined for imaging vasculature and tumors [39], gene therapy, drug delivery [40], tracing of labeled cells [41], thermal ablation of tumors via magnetic heating [42], and organ preservation [43]. Within the last decade, the Food and Drug Administration (FDA)’s approval of ferumoxytol (Feraheme) to nurse patients with iron deficiency and chronic kidney disease highlighted the clinical applicability of SPIONs in therapy [44]. It was reported that patients tolerated up to 510 mg Fe/injection, with subsequent growth in haemoglobin level post-injection [45]. No serious adverse events were observed from the study that was reported in 396 US patients who received a total of 570 intravenous (IV) injections of SPION therapy.

## 3. Multimodal Cancer Theranostics

There are several known molecular imaging modalities such as MRI, single-photon emission computerized tomography (SPECT), and positron emission tomography (PET); however, none of them are perfect and adequate to acquire all the necessary information for a particular question [46]. For instance, it is challenging to quantitatively determine fluorescence signal in vivo, specifically in deep tissues; although the use of MRI would render high resolution, it suffers from low sensitivity, whereas imaging methods relying on radionuclide show very high sensitivity but poor resolution. Therefore, the blend of multiple molecular imaging techniques provides a symbiotic advantage as compared to separate individual modalities. Thus, this review describes the combination of magnetite NPs with rhenium(I) tricarbonyl complexes. Due to the inherently low sensitivity of MRI, exogenous contrast agents such as the magnetic Fe_3_O_4_ NPs (induces higher magnetic fields, 4.7–14 T in small animal models) are incorporated to enhance sensitivity and to obtain data for a much longer period. In this instance, a crystalline Fe_3_O_4_ core is commonly incorporated into a polymer coating material such as dextran or poly(ethylene glycol) PEG for its use as an MRI contrast agent [47]. As a result, the existence of thousands of iron atoms in each particle will produce a high T_2_ relaxivity [48].

Additionally, Fe_3_O_4_ NPs can be attached to a radionuclide such as ^187/188^Re to dramatically amplify the signal, enhance receptor-binding affinity, improve the detection sensitivity and quantify imaging, which is only true if the radioisotope remains bound to the NP. ^187/188^Re isotopes form part of the first radionuclides that were put on trial for NP-based radiotherapy. Amongst them, ^188^Re has interestingly been examined for magnetically targeted radiotherapy [49,50]. For instance, when the surface of silica-coated Fe_3_O_4_ NPs is labeled by ^188^Re with >90% labeling yield and good in vitro stability, the radioisotope uptake in the tumor is enhanced as a magnetic field is simultaneously applied above the tumor area [51,52]. Liang et al. (2007) reported the successful attachment of amino-functionalized superparamagnetic Fe_3_O_4_ NPs with a humanized monoclonal antibody targeted for liver cancer cells. They then radiolabelled with ^188^Re and consequently, due to their size (between 10 and 15 nm in diameter), these NPs were expected to have high uptake in the reticuloendothelial system (RES), e.g., liver, and to uplift magnetically targeted radiotherapy for the treatment of liver cancer [53].

**Scheme 1:**
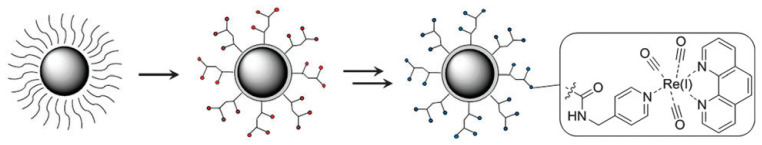
Design of the magnetoluminescent iron oxide nanoparticles. Red and blue circles represent hydrophilic Fe_3_O_4_ NPs with multiple acid functions and the luminophore, respectively [22].

Radiolabelling of magnetic NPs creates a potential bimodal contrast agent for MRI and optical imaging; hence, a few examples attributed to the combination of magnetic Fe_3_O_4_ NPs and Re(I) tricarbonyl complexes are illustrated which are in line with the aim of this review. However, other general examples concerning the potential application of Re(I) tricarbonyl complexes functionalized with other types of NPs are also shown. A siloxane luminophore is normally used to functionalize the surface of magnetite to yield water-dispersible Fe_3_O_4_-NPs (as illustrated in Scheme 1). This is a convenient way because it produces a biocompatible, inert, and permanent shell that is commonly known for its diverse functionalities. Additionally, it creates a thin layer of functionalized siloxanes around the Fe_3_O_4_ NPs which forms an appropriate scaffold for linking Re(I) tricarbonyl complexes [54,55]. In this instance, oleate functionalized Fe_3_O_4_ NPs are treated with N-(trimethoxysilylpropyl) ethylenediamine triacetic acid trisodium salt to acquire hydrophilic Fe_3_O_4_ NPs with multiple acid functions. This is followed by a multistep preparation with picolylamine, which reacts with the free acid of the NPs to produce a peptide bond with the metal.

**Scheme 2:**
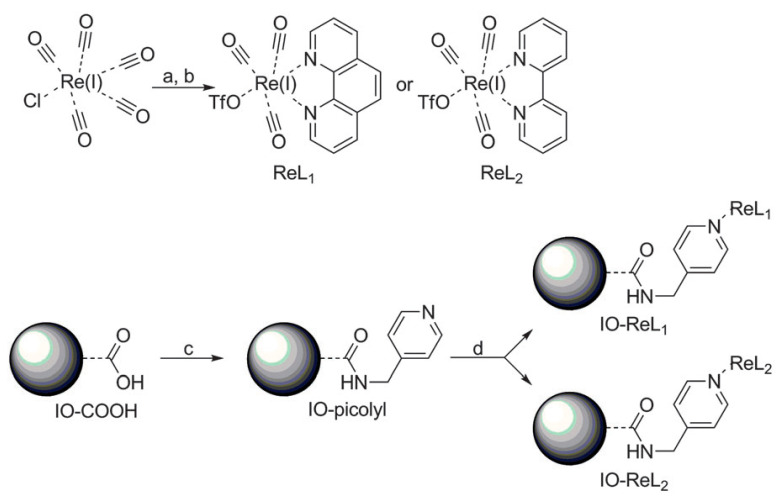
Synthetic procedure for the IO-ReL1 and IO-ReL2 molecules. (a) 2 eq. 1,10-Phenanthroline (L1) or 2,2-bipyridine (L2), 1 eq. ClRe(CO)_5_, benzene, 333 K, 5 h. (b) AgOTf, THF/MeCN, 16 h. (c) H_2_O/THF, HCl, 1-ethyl-3-(3-dimethylaminopropyl)carbodiimide (EDC), N-hydroxysuccinimide (NHS), sonicate 30 min. (d) H_2_O/CAN, NaHCO_3_, 223 K, 17 h [22].

Interestingly, the Re(I) tricarbonyl complexes (illustrated in Scheme 2) possess potential luminescent properties between the 590 and 620 nm region of the electromagnetic spectrum; hence, they have been identified as the best candidates to be used as OI contrast agents for cancer theranostics [56,57]. This potential of the Re(I) tricarbonyl complex antenna structure has been found useful due to its high affinity towards the pyridine ligands, whilst keeping the Fe_3_O_4_ NPs as small as possible so that the benefits of T_1_ and T_2_ contributions can be useful for MRI applications [58,59,60].

## 4. Phosphorescence Transition Metal Complexes for Tumor Diagnosis

Several transition metal complexes exhibit different types of excited states depending on the metal centres, the triplet-state energy levels of the ligand, and the local environment. These excited states include metal-to-ligand-charge-transfer (MLCT), intraligand-charge-transfer (ILCT) as well as ligand-to-ligand-charge-transfer (LLCT), and these are mostly found in heavy-metal complexes. However, the MLCT state is commonly seen in transition metal complexes with *d*^6^ and *d*^8^ configurations; therefore, MLCT is in charge of phosphorescence emission [61].

Phosphorescence is referred to as the process whereby energy is absorbed by a substance and subsequently released slowly in the form of light. Phosphorescent transition metal complexes (PTMCs), such as Ru(II), Re(I), Ir(III), and Au(I) complexes, show potential as phosphorescent imaging agents. Thus, they are versatile and form a dynamic scaffold for the growth of tumor diagnostic probes due to their advantageous photophysical properties such as large Stokes shifts, long luminescent lifetimes, and resistance to photo-bleaching [61,62]. Furthermore, by varying the ligands around these types of complexes (PTMCs), their photo-physical properties can be easily tuned [63]. For instance, the emission spectra will be shifted into the near-infrared radiation when there is an addition of an extensive electronic system in the co-ligands. This is more favored for biological imaging because near-infrared rays penetrate through into deeper tissues within the range of 750–950 nm [64].

**Figure 2:**
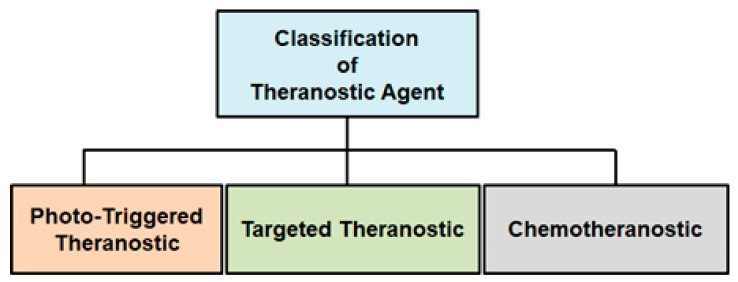
Design strategies and mechanism of different theranostic approaches.

Additionally, the triplet-excited state of PTMCs confers a long-lived phosphorescence (hundreds of nanoseconds (ns) to microseconds (μs), much larger than those of organic fluorophores) with a greater Stokes shift [65,66]. Stokes shift is the distinction between the wavelength at which a molecule emits light and the wavelength at which it was excited. These unique properties permit facile differentiation of the PTMC signal from a highly auto-fluorescent background and also neglect the self-quenching of fluorescence that is displayed by some organic dye molecules [67]. This section outlines the use of phosphorescent Re(I) tricarbonyl complexes for cancer diagnostic applications. The use of α-diimine ligand in the *fac*-[Re(CO)_3_(X)(α-diimine)] (X = halide) structure exerts a powerful influence on the MLCT properties. The application of *fac*-[Re(CO)_3_(X)(α-diimine)] complexes is advantageous because they allow easy synthesis and give some of the earliest insights into the applications of molecular metal complexes. The vigorous anticancer activity of the existing metal-based chemotherapeutic drugs gives rise to a range of unwanted adverse side effects due to their non-specific distribution throughout the body. Nonetheless, the systematic administration and pharmacokinetics of anticancer drugs as well as the precision of therapeutic drug delivery can be enhanced by the combination of therapeutic and diagnostic approaches into a single ‘‘theranostic” modality. The type of therapeutic modality classifies the anticancer drugs that should be used depending on the kind of therapy, shown in Figure 2.

PTMC-based theranostic agents generally comprise two main constituents, namely: metal complex core and a targeting ligand. On the other hand, theranostic agents based on non-emissive transition metal-based drugs generally need three components, namely: an imaging luminophore, a metal-based pro-drug as well as a targeting ligand. Most importantly, a transition metal complex can be thought to comprise separate modules that each possess different functionalities depending on the type of theranostic method used. For example, the non-emissive platinum pro-drug, such as *cis*-platin, can be coordinated with extended ligands that behave as the signal transducer and targeting moiety for chemo-theranostic imaging. In contrast, with photodynamic therapy, the complex is emissive for optical imaging. The metal centre reacts as a scaffold for producing reactive radicals for therapeutic aim, while the ligands act as the targeting moiety. Therefore, with the growing interest in organometallic chemistry, several transition metal complexes-based theranostic agents, such as *fac*-[Re(CO)_3_(X)(α-diimine)] (X = halide), have been synthesized with enhanced selectivity, permeability, efficacy, retention, and cellular uptake efficacy.

### 4.1. Luminescent Rhenium(I) Tricarbonyl Complexes

The exploitation of *facial* rhenium(I) tricarbonyl α-diimine complexes dates back to the 1970s. Their chemical properties have attracted much attention because of their useful photo-physical attributes. Most recently, they have been widely applied as imaging agents in human cell lines due to their biological stability [56,62,68,69,70]. These types of Re(I) tricarbonyl complexes with the general formula *fac*-[Re(CO)_3_(*N*,*N’*)X]^n+^, (where *N*,*N’* = 1,10-phenanthroline (phen) or 2,2 –bipyridine (bpy) X = anionic or neutral monodentate ligand and *n* = 0 or 1, respectively), have been widely studied due to their distinctive luminescent properties [71]. Additionally, the existence of a single electron-acceptor α-diimine ligand, which negates the problem of localization of the excited electron normally occurring for polypyridine ruthenium(II) complexes, makes these complexes extremely interesting also for basic photo-physical studies [72]. The Re(I) tricarbonyl -diimine complexes display *d* Re → *N,*N’* MLCT absorptions, which are similar to other *d*^6^ transition metal complexes. These complexes show relatively high molar absorptivity (ε = 104 cm^−1^.M^−1^) and moderately long-lived excited states (typically 0.1–1 s in solution at room temperature). During optical excitation most of these species exhibit intense and unstructured emission in solution, centred at approximately 600 nm, which emanates from the MLCT excited states that are mainly of triplet character. According to Villegas et al. (2005) [73], very high photoluminescence quantum yields (up to 0.8°) can be acquired for cationic species, whereas those of neutral species normally do not surpass 0.05 [74].

**Figure 3:**
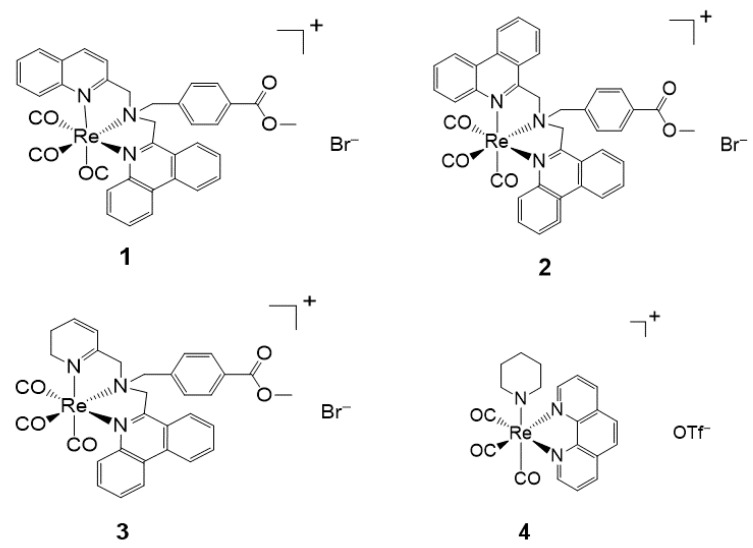

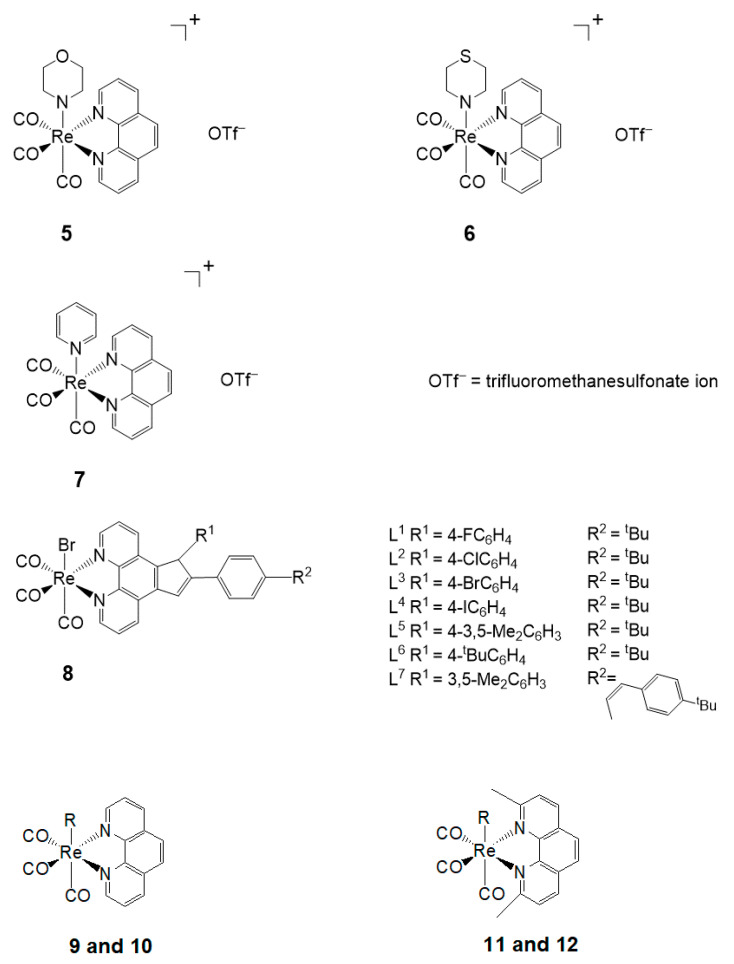

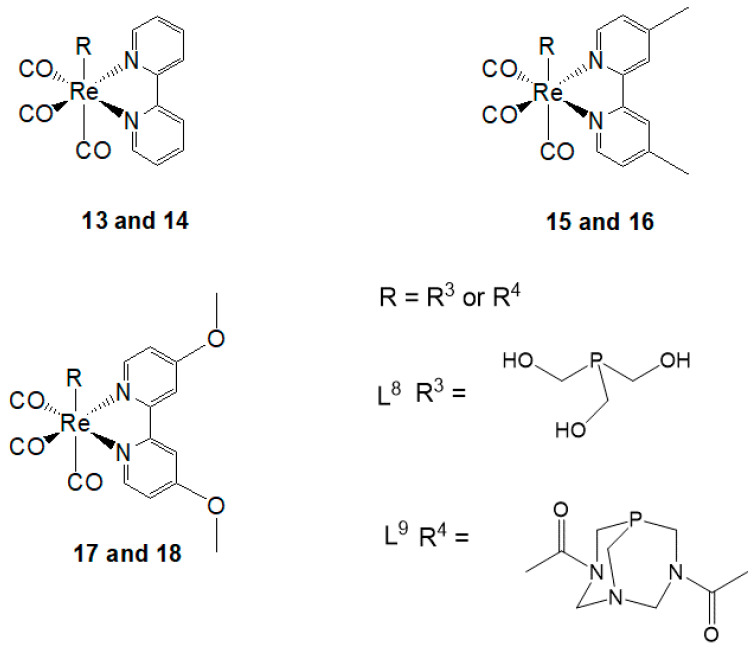
Re(I) tricarbonyl complexes with favorable photophysical properties. Complexes **4**–**17** also show biological activities.

The novel Re(I) tricarbonyl complexes 1–18 (see Figure 3) possess favorable photophysical properties (i.e., emission lifetimes (τ), percentage quantum yields (Φ), emission energy (λ_max_), as shown in Table 2) at a given maximum wavelength (λ_max_). Significantly, their favorable luminescence behavior can be displayed in various solutions such as degassed acetonitrile, chloroform, and air-equilibrated water, however small these variations are in the different solvents. These beneficial luminescence properties are further highlighted by the successful application of the reported complexes (see Table 2) as imaging agents.

### 4.2. Chemo-Theranostic

Chemotherapy is an effective type of cancer treatment utilizing chemotherapeutic agents, which mostly function by impairing mitosis (a division of cells into two daughter cells) in rapidly dividing cancer cells. Transition metal complexes were found to have greater use in the development of chemotherapeutic agents because of their DNA alkylation and/or intercalation abilities. For instance, platinum-based alkylating agents such as *cis*platin are exceptionally effective against different types of cancers, for instance, testicular and ovarian cancers. However, their small size and square planar geometry result in them achieving poor site exploitation at the double-helix level. These limitations instigated the growth of new chemotherapeutic techniques with alternative metals and geometries such as Tc and Re [79].

### 4.3. Cellular Imaging

Many photophysical properties of luminescent transition metal complexes, for example, large Stokes shifts, long luminescent lifetimes, and resistance to photo-bleaching in addition to low toxicity and good uptake, make them better candidates to be used as cell imaging agents [80]. Therefore, several mononuclear rhenium(I) tricarbonyl complexes with a variety of charges and degrees of hydrophobicity have been synthesized and utilized as luminophores in fluorescence cell imaging [81]. On the other hand, chemical groups have been presented in the ligand sphere to interact with or bind to specific biological targets [82]. Additionally, the localization of the excited state of *fac*-[Re(CO)_3_(bpy)(X)], (X = halide) complexes on the distinctive bipyridine chromophore [83,84] make easier modifications to permit a response to the environment. The emission from these types of systems is especially sensitive to the local surrounding [85], that involves hydrophobicity of the environments; thus, they can be further used as bio-sensing probes [86].

## 5. Biological Studies

**Figure 4:**
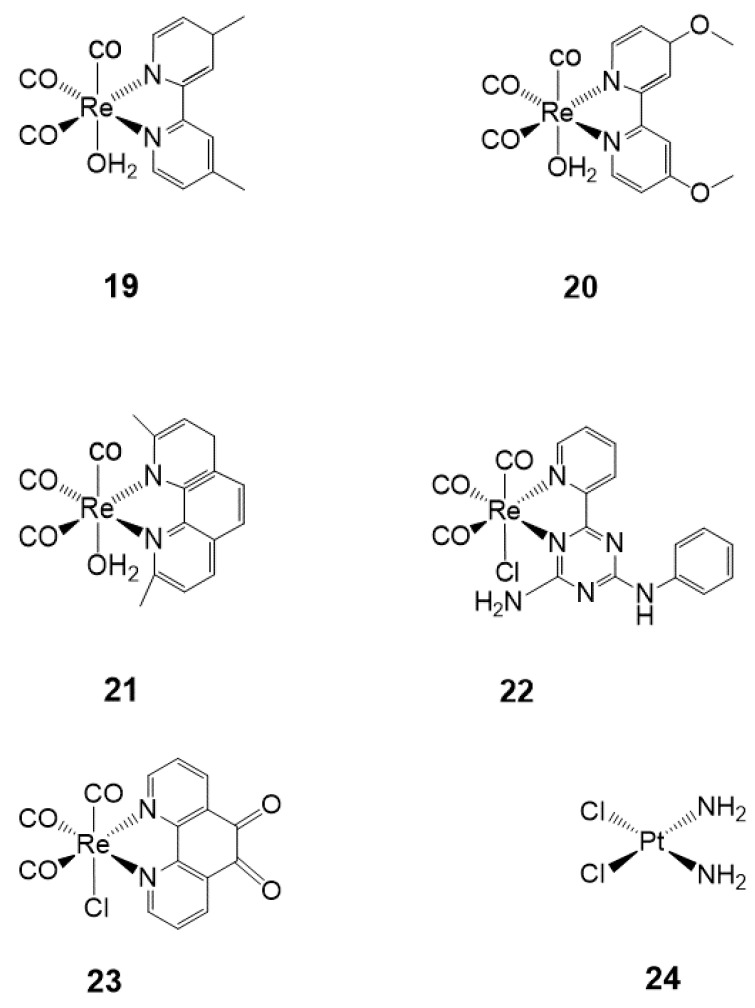
Biologically active Re(I) tricarbonyl complexes 19–23 as compared to cisplatin 24.

Although cisplatin is a clinically approved drug for cancer therapy, platinum resistance remains the primary concern due to genetic and epigenetic changes of various cellular routes [87]. Hence, several studies currently focus on fighting against resistance and consequently substituting these old drugs. Recently, several studies involving in vitro testing of Re(I) tricarbonyl complexes with the focus on the development of novel and target-specific chemotherapeutic drugs have been reported [87,88]. Herein, the cytotoxicity of a variety of biologically active Re(I) tricarbonyl complexes is explained in different cell cultures. To comprehend the extent of cancer drug cytotoxicity, in vitro applications in different cell lines are performed [89]. Cytotoxicity in cells is described as the inhibitory concentration (IC_50_) needed for a specimen or complex to kill 50% of the cell population. IC_50_ values are used to express cytotoxicity, which is determined as the mean percentage increase in comparison to the untreated control. Furthermore, to evaluate the cytotoxic ability of a complex, selectivity index (SI) is applied by measuring the ratio of IC_50_ of normal cells to the IC_50_ of cell death population in cancer cells [90,91]. The SI values are indicative of whether a complex is noncytotoxic or not (i.e., the greater the SI value, the more selective a compound is). An SI value of > 2 shows that a compound has selective cytotoxic activity; however, an SI value of < 2 indicates the general cytotoxicity towards cells [89]. Additionally, cellular systems obtained from cancerous tissues are frequently utilized to examine the cytotoxicity of new complexes, which is done by comparing the effect of the compounds on the tissues. Most importantly, the right concentration (µM) of test materials determines whether a particular compound is an active anticancer agent or inactive antiproliferation of cancer cells [92]. Table 3 shows different Re(I) tricarbonyl complexes 19–24 that have been tested and found to possess some cytotoxic activity against their respective cell lines: HeLa, HT-29, PT-45, HepG2, U-2 OS, A2780, CP70, etc. However, according to Knopf et al. (2017), the use of different cell lines may result in inconsistencies in some observed biological properties of complexes [93]. Furthermore, the review by Haase et al. (2019), emphasized that ligands may also play a significant role in the cytotoxicity of Re(I) tricarbonyl complexes [94]. A comparative Table 3 illustrates the active concentrations of the Re(I) tricarbonyl complexes to that of cisplatin (see Figure 4) as the reference active anticancer drug.

## 6. Concluding Remarks

Magnetic NPs, particularly SPION crystals, have been a field of active research for pharmaceutical application. The successful clinical translation of these NPs for use in magnetic resonance (MR) contrast imaging, cancer treatment through hyperthermia, and sentinel lymph node (SLN) mapping stand as clear examples of the promise of nanotechnology to modify clinical practice and lead to enhanced patient care. Furthermore, the presence of *d*-block metal centres, specifically Re(I) tricarbonyl complexes, enables transition metals to set up new electronic states, which result in characteristic photophysical and photochemical properties that are essentially different from those of fluorescent substances such as organic dyes, lanthanide chelates, and quantum dots. Thus, the high photostability, long emission lifetimes, large Stokes shifts, inter/intramolecular energy/electron transfer, and the photogeneration of reactive oxygen species, make Re(I) tricarbonyl complexes promising candidates for the design of specific cell imaging reagents for biological applications. This review outlined the synergistic effect arising from the combination of magnetic NPs with luminescent Re(I) tricarbonyl complexes which results in excellent MRI-OI probes for nanomedicine in cancer theranostics.

## Figures and Tables

**Table 1:** The categories of radionuclides used as therapeutic and diagnostic agents [5].

Radionuclide	Half-Life (t_1/2_)	Emission	E_max_
^188^Re	89.2 h	β,γ	1.07 MeV
^186^Re	17.0 h	β,γ	2.12 MeV
^99m^Tc	6.0 h	γ	140 KeV

**Table 2:** Photophysical properties of Re(I) tricarbonyl complexes **1**–**3** [75], **4**–**7** [76], **8** (L^1^–L^7^) [77] and **9**–**18** [78].

Complexes	Solvent	Emission			Absorption
		τ (μs)	Φ (%)	λ_max_ (nm)	λ_max_ (nm)/ ε (L.mol^−1^. cm^−1^)
**1**	Acetonitrile	0.46	0.12 ± 0.03	580	310 (13,400); 350 (10,600)
**2**	Acetonitrile	0.45	0.12 ± 0.01	609	310 (9700); 350 (7300)
**3**	Acetonitrile	0.58	0.18 ± 0.01	593	310 (13,100), 350 (8200)
**4**	Air-equilibrated aqueous	0.31	1.66 ± 0.06	560	225 (31,300 ± 1300) 257 (18,200 ± 800)
**5**	Air-equilibrated aqueous	0.52	2.14 ± 0.16	560	24 (30,700 ± 1900)
**6**	Air-equilibrated aqueous	0.47	2.38 ± 0.31	560	22 (32,300 ± 1000)
**7**	Air-equilibrated aqueous	0.62	3.09 ± 0.30	545	23 (35,300 ± 600)
**8** L^1^	Chloroform	0.170	-	577	276, 362, 410
**8** L^2^	Chloroform	0.153	-	585	277, 339, 423
**8** L^3^	Chloroform	0.143	-	581	277, 338, 423
**8** L^4^	Chloroform	0.141	-	586	277, 337, 423
**8** L^5^	Chloroform	0.158	-	582	282, 349, 423
**8** L^6^	Chloroform	0.162	-	580	279, 352, 419
**8** L^7^	Chloroform	0.185	-	586	288, 342, 423
**9**	Air-equilibrqted Phosphate-Buffered Saline (pH 7.40)	1.5	5.1 ± 1.1	528	226 (36,800 ± 1300), 275 (26,800 ± 900), 322 (6400 ± 200), 366 (3600 ± 100)
**10**	Air-equilibrqted Phosphate-Buffered Saline (pH 7.40)	1.9	10.7 ± 0.6	516	225 (41,600 ± 7300), 275 (27,200 ± 4900), 323 (5700 ± 900), 367 (3200 ± 400)
**11**	Air-equilibrqted Phosphate-Buffered Saline (pH 7.40)	1.0	4.5 ± 0.4	518	228 (34,600 ± 1200), 286 (23,400 ± 800), 308 (11,900 ± 400), 372 (2100 ± 70)
**12**	Air-equilibrqted Phosphate-Buffered Saline (pH 7.40)	2.0	7.2 ± 0.2	507	227 (36,800 ± 6800), 285 (23,800 ± 700), 309 (11,500 ± 300), 373 (2100 ± 50)
**13**	Air-equilibrqted Phosphate-Buffered Saline (pH 7.40)	0.4	6.1 ± 1.7	536	222 (18,900 ± 200), 249 (20,100 ± 1900), 308 (10,700 ± 400), 318 (12,500 ± 400), 343 (3600 ± 700)
**14**	Air-equilibrqted Phosphate-Buffered Saline (pH 7.40)	0.6	9.1 ± 3.0	528	246 (22,600 ± 500), 308 (107,00 ± 200), 319 (13,000 ± 300), 345 (3400 ± 100)
**15**	Air-equilibrqted Phosphate-Buffered Saline (pH 7.40)	0.4	6.5 ± 2.0	528	252 (24,000 ± 3000), 304 (11,800 ± 1500), 315 (13,400 ± 1700), 338 (4600 ± 500)
**16**	Air-equilibrqted Phosphate-Buffered Saline (pH 7.40)	0.6	11.5 ± 3.9	518	250 (82,00 ± 4300), 305 (12,800 ± 2000), 315 (14,900 ± 2300), 339 (4000 ± 400)
**17**	Air-equilibrqted Phosphate-Buffered Saline (pH 7.40)	0.3	3.4 ± 1.1	537	223 (33,900 ± 1000), 251 (30,500 ± 900), 303 (8800 ± 300), 337 (4500 ± 80)
**18**	Air-equilibrqted Phosphate-Buffered Saline (pH 7.40)	0.4	7.1 ± 3.0	527	224 (41,400 ± 2000), 251 (34,100 ± 1900), 303 (9600 ± 500), 332 (4700 ± 300)

**Table 3:** IC_50_ values of Re(I) tricarbonyl complexes **4**–**7** [76], **8** (L^1^–L^7^) [77], **9**–**18** [78], **19**–**22** [93], **23** [95], as compared to the cisplatin drug **24** [96,97,98,99,100].

Complex	Cell Lines	Cell Line Target Description	IC_50_ (µM)	Cytotoxicity (IC_50_)
**4**	HeLa	Cervical cancer cell	>164	Inactive (<100 μM)
**5**	HeLa	Cervical cancer cell	>185	Inactive (<100 μM)
**6**	HeLa	Cervical cancer cell	36 ± 3	Modest activity (51 μM)
**7**	HeLa	Cervical cancer cell	51 ± 5	Modest activity (36 μM)
**9**	HeLa	Cervical cancer cell	26.4 ± 9.2	Active
**10**	HeLa	Cervical cancer cell	5.9 ± 1.4	Active
**11**	HeLa	Cervical cancer cell	9.6 ± 4.2	Active
**12**	HeLa	Cervical cancer cell	19.2 ± 2.9	Active
**14**	HeLa	Cervical cancer cell	14.9 ± 3.2	Active
**16**	HeLa	Cervical cancer cell	60.3 ± 18.2	Active
**17**	HeLa	Cervical cancer cell	68.0 ± 4.3	Active
**18**	HeLa	Cervical cancer cell	24.3 ± 9.1	Active
**19**	A2780	Human ovary epithelial cell, ovarian endometrioid adenocarcinoma.	3.5 ± 2.8	Active
**20**	A2780	Human ovary epithelial cell, ovarian endometrioid adenocarcinoma.	2.2 ± 1.8	Active
**21**	A2780	Human ovary epithelial cell, ovarian endometrioid adenocarcinoma.	2.2 ± 0.2	Active
**22**	HT-29	Human colon epithelial cell, adenocarcinoma.	<250	Active
	PT-45	Human pancreas epithelial cell, adenocarcinoma.	<250	Active
**23**	T98G	Human brain fibroblast, glioblastoma.	>50	Active
	PC3	Human prostate epithelial cell, adenocarcinoma.	>50	Active
**24**	HT-29	Human colon epithelial cell, adenocarcinoma.	32.6 ± 0.7	Active
	PT-45	Human pancreas epithelial cell, adenocarcinoma.	2.2 ± 0.3	Active
	HepG2	Human liver epithelial cell, hepatocellular carcinoma.	10.5 ± 0.5	Active
	T98G	Human brain fibroblast, glioblastoma.	6.45 ± 1.64	Active
	PC3	Human prostate epithelial cell, adenocarcinoma.	2.19 ± 0.11	Active
	A2780	Human ovary epithelial cell, ovarian endometrioid adenocarcinoma.	0.23 ± 0.07	Active
	HeLa	Cervical cancer	6.6 ± 0.7	Active

## Data Availability

Not applicable.

## Abbreviations

NP(s)	Nanoparticle(s)
Fe_3_O_4_	Ferric Oxide or Magnetite
Fe_2_O_3_	Ferrous oxide or Hematite
DNA	Deoxyribonucleic Acid
DMSO	Dimethylsulfoxide
MCF-7	Michigan Cancer Foundation-7
Ag	Silver
Ru	Ruthenium
Ir	Iridium
^99m^Tc	Technetium-99m
MRI	Magnetic Resonance Imaging
FM	Fluorescence Microscopy
Mm	Mega-meter
W	Watt
^186^Re	Rhenium(I) 186
^188^Re	Rhenium(I) 188
α	Alpha particle
β	Beta particle
γ	Gamma-ray
t_1/2_	Half-life
E_max_	Maximum Energy
MeV	Mega Electron Volt
KeV	Kilo Electron Volt
^1^O_2_	Singlet Oxygen
CO	Carbon monoxide
HeLa	Henrietta Lacks
N	Nitrogen
LED	Light Emission Diode
O	Oxygen
M	Metals
XRD	X-ray Diffraction
SLN	Sentinel Lymph Node
TEM	Transmission Electron Microscopy
L	Ligand
OI	Optical Imaging
T_1_	Longitudinal Relation Time
T_2_	Transverse Relation Time
SPION	Superparamagnetic Iron Oxide Nanoparticles
OI	Optical Imaging
Br^−^	Bromide
Cl^−^	Chloride
H_2_O	Dihydrogen Monoxide
ILCT	Intraligand-Charge-Transfer
MLCT	Metal-to-Ligand-Charge-Transfer
LLCT	Ligand-to-Ligand-Charge-Transfer
H	Hour(s)
PTMCs	Phosphorescent Transition Metal Complexes
